# Medical Opinion and Movement

**Published:** 1908-12-19

**Authors:** 


### Medical Opinion and Movement.

OUR knowledge of the digestive processes has been considerably enhanced by the method of observation by means of the $x$-rays, the food being rendered visible by admixture with bismuth carbonate, and reference has been frequently made in these columns to the experimental work that has been carried out on these lines. Dr. Magnus has recently investigated the action of morphia upon the alimentary tract in this way, and the results he has obtained are somewhat surprising. He finds that the retarding influence of the morphia upon the progress of the food makes itself felt chiefly in the stomach. The food remains for several hours in the cardiac end of the stomach, but when once it reaches the pylorus its further passage is almost the same as under normal conditions. It appears, therefore, that the morphia inhibits the natural peristaltic churning action of the cardia and so prevents the proper digestion of the food. It has only a feeble action on the small intestine and does not affect the peristalsis of the large intestine at all. It follows from this that diarrhœa provoked by such purgatives as castor oil or senna, which act directly on the intestine, is not much influenced by morphia.

THE extensive researches on the action of ferments and serums which are being carried out at the Rudolf Virchow Hospital at Berlin, under the superintendence of Professor Bier, have led to the discovery that solutions of certain ferments, especially trypsin, have the effect of causing absorption of purulent collections, notably in tuberculous conditions. The result is probably due to the peptonising effect of the ferment upon the albuminoids in the exudate. A one per cent. solution of trypsin in normal saline, carefully sterilised, is injected into the tuberculous focus, or, in the case of ulcerating surfaces, they are irrigated with the solution and afterwards covered with dry gauze. The injections and irrigations are repeated at varying intervals. One or two cubic centimetres of the solution are injected at a time. No local or general disturbance results from the injections. The method has been used in cases of suppurating glands, subcutaneous abscesses, and tuberculosis of the bones and joints. As a result of these injections there is absorption of the purulent exudates and a rapid development of healthy granulation tissue. Sinuses and ulcerating surfaces tend to dry up and heal, and the general condition of the patient improves. Drs. Jochmann and Bätzner are the authors of this line of research, and although they hesitate to pronounce definitely upon the value of the method of treatment, they are of opinion that clinical observations so far are sufficiently encouraging to justify a further pursuit of the treatment.

SOME interesting experiments have been carried out by Dr. Hofbauer on the production of abortion by the administration of cholesterin. The cells of the fœtus, and especially those of the placenta, are known to contain a large proportion of lecithin; and it is supposed that any interference with the normal production of this lecithin acts deleteriously upon the development of the fœtus. Acting upon these ideas and upon the assumption of a physiological antagonism between lecithin and cholesterin, Dr. Hofbauer endeavoured to obtain experimental abortion in pregnant rabbits by giving cholesterin by the mouth mixed with the food. He administered ten cubic centimetres of a 5-per-cent. solution of cholesterin three times a day. One animal in the middle of pregnancy aborted on the fourth day, and was delivered of two dead fœtuses. In the case of two other animals who were nearly at full term abortion did not take place. The uterus was opened therefore after a few days' treatment, and the fœtuses were found dead. Examination showed maceration and considerable degenerative changes in the tissues of the fœtuses and in the placentæ. It appears, therefore, that cholesterin is able to produce such degenerative changes in the placenta as to seriously interfere with its function, and in consequence cause the death of the fœtus. Dr. Hofbauer thinks these changes are due to destruction of the lecithin. Professor Wassermann has found in the course of his researches with syphilitic serum that there are certain toxic substances in the serum which are able to decompose lecithin, and Dr. Hofbauer suggests that the maceration of the fœtus and occurrence of abortion in syphilis may be due to this decomposing action of the syphilitic virus in the mother on the lecithin of the fœtus.

THE Sleeping Sickness Bureau has recently published a Quarterly Report on the progress of the segregation camps and medical treatment of sleeping sickness in Uganda. One new drug, Soamin (sodium-amino-phenyl-arsenate—Burroughs Wellcome and Co.), has been tried, and Dr. van Someren reports favourably on it. The results of its administration, in his opinion, are as good as those obtained by atoxyl, the different samples are very uniform, and its cost is only one-third of the latter. Further, it is supposed not to give rise to toxic symptoms; but, of course, subsequent trials may explode this idea. The report gives the death-blow to the idea that atoxyl is a general and permanent cure for sleeping sickness; all, apparently, that can be said of it and allied drugs is that it has greatly prolonged life in many cases, and may, even in some patients under observation now, have acted as a cure. This is very disappointing news, and the gloom is not dissipated by the further fact that the combined treatment with atoxyl and mercury—a treatment proposed by the Liverpool School of Tropical Medicine, and believed by them to be specific—has also turned out a failure. Antimony apparently comes under the same category, so it is still open for someone to discover a drug that will really completely destroy the trypanosomes in the body, and so produce a permanent cure.

